# Data supporting the co-expression of *PDHA1* gene and of its paralogue *PDHA2* in somatic cells of a family

**DOI:** 10.1016/j.dib.2016.08.029

**Published:** 2016-08-20

**Authors:** Ana Pinheiro, Maria João Silva, Hana Pavlu-Pereira, Cristina Florindo, Madalena Barroso, Bárbara Marques, Hildeberto Correia, Anabela Oliveira, Ana Gaspar, Isabel Tavares de Almeida, Isabel Rivera

**Affiliations:** a Metabolism & Genetics Group, Research Institute for Medicines (iMed.ULisboa), Faculty of Pharmacy, Universidade de Lisboa, Portugal; b Department of Biochemistry and Human Biology, Faculty of Pharmacy, Universidade de Lisboa, Portugal; c Department of Human Genetics - Molecular Cytogenetic Unit National Institute of Health Doctor Ricardo Jorge, I.P., Lisboa, Portugal; d Department of Medicine, Hospital Santa Maria, Lisboa, Portugal; e Department of Pediatrics, Hospital Santa Maria, Lisboa, Portugal

**Keywords:** Pyruvate dehydrogenase, Complex gene expression, Testis-specific gene

## Abstract

This article presents a dataset proving the simultaneous presence of a 5′UTR-truncated *PDHA1* mRNA and a full-length *PDHA2* mRNA in the somatic cells of a PDC-deficient female patient and all members of her immediate family (parents and brother).

We have designed a large set of primer pairs in order to perform detailed RT-PCR assays allowing the clear identification of both *PDHA1* and *PDHA2* mRNA species in somatic cells. In addition, two different experimental approaches were used to elucidate the copy number of *PDHA1* gene in the patient and her mother.

The interpretation and discussion of these data, along with further extensive experiments concerning the origin of this altered gene expression and its potential therapeutic consequences, can be found in “Complex genetic findings in a female patient with pyruvate dehydrogenase complex deficiency: null mutations in the *PDHX* gene associated with unusual expression of the testis-specific *PDHA2* gene in her somatic cells” (A. Pinheiro, M.J. Silva, C. Florindo, et al., 2016) [1].

**Specifications Table**

**Table:** 

Subject area	*Biology*
More specific subject area	*Molecular Genetics*
Type of data	*Tables, figures*
How data was acquired	*Agarose gel electrophoresis after RT-PCR analyses quantitative real time PCR, microarray analyses, in silico analyses (BLAST software)*
Data format	*Raw, analyzed*
Experimental factors	*Genomic DNA and total RNA isolated from whole blood samples and fibroblast cultures*
Experimental features	*Genomic DNA was amplified by quantitative real time PCR and microarray analyses. Total RNA was reverse transcribed and amplified by semi-quantitative RT-PCR and by quantitative real time PCR using TaqMan assays. Alignment of sequences was performed using the BLAST software.*
Data source location	*Lisboa, Portugal*
Data accessibility	*Data provided within the manuscript and available in public databases (NCBI) in case of sequence alignment: GenBank accession numbers GenBank:*NM_000284.3*(PDHA1) and GenBank:*NM_005390.4*(PDHA2)*

**Value of the data**

• These data, reporting on *PDHA2* gene expression in somatic cells, may trigger new research related to the activation of a paralogue gene as a therapeutic target to loss-of-function mutations.
• Data revealing the co-existence of both *PDHA1* and *PDHA2* mRNAs in somatic cells will be useful for future experiments addressing the impact between both isoforms in the assembly of a fully functional PDC.
• Data concerning gene copy number may assist the choice of the underlying methodology.
• These dataset may contribute for designing further experiments aiming the development of alternative therapies for metabolic disorders.

## 1 Data

The E1 rate-limiting enzyme of pyruvate dehydrogenase complex (PDC) is a heterotetramer (α_2_β_2_) and its α subunit is encoded by *PDHA1* gene, located in X chromosome and presenting ubiquitous expression in somatic tissues. Nevertheless a paralogue gene exists, *PDHA2*, which is located in chromosome 4 and expressed only in spermatocytes and spermatids [2].

**Fig. 1:**
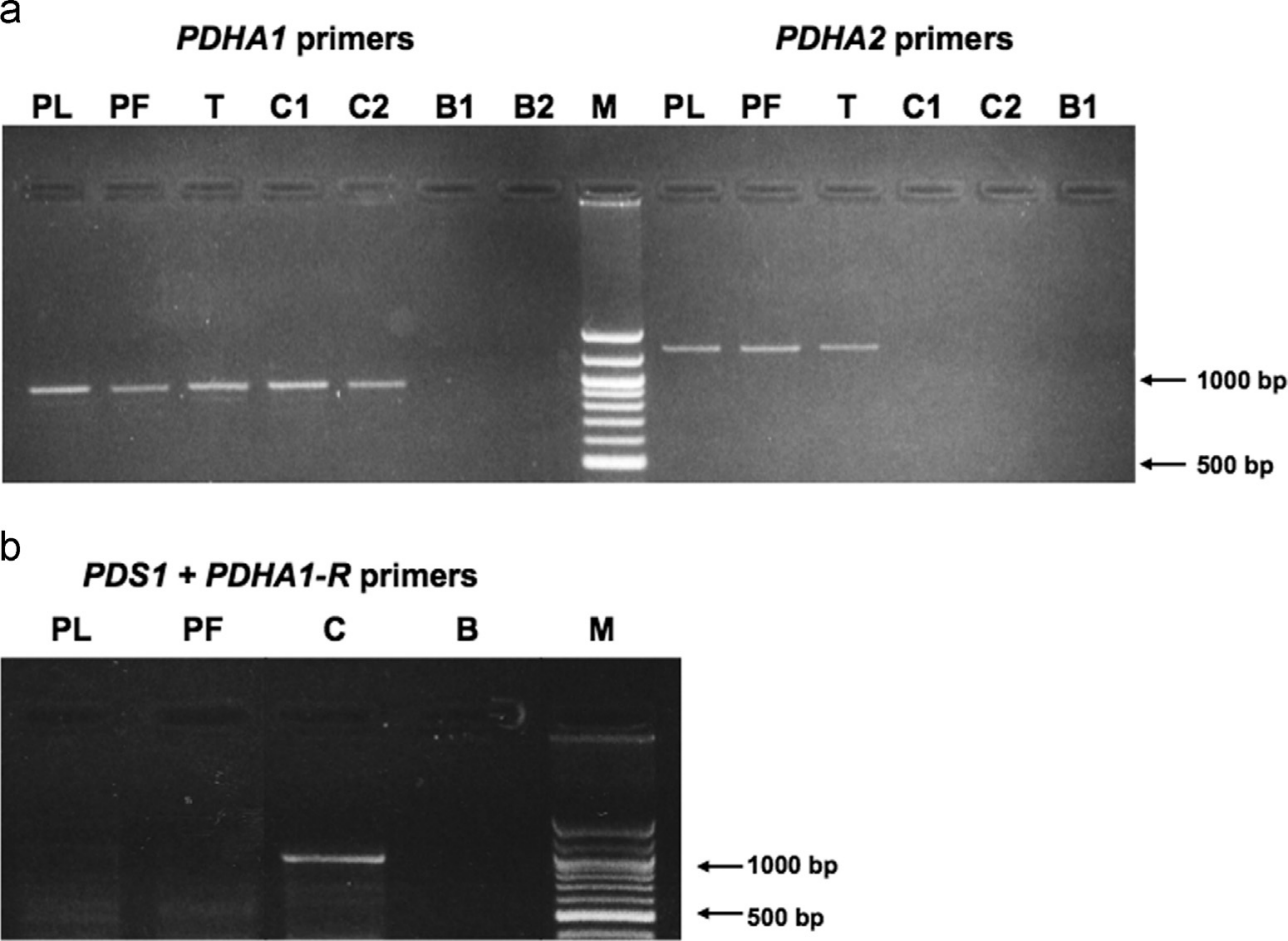
RT-PCR analyses of PDH E1α transcripts. (a) Using *PDHA1* and *PDH2* specific primers. PL - patient lymphocytes; PF - patient fibroblasts; T - whole testis tissue; C1 and C2 - control lymphocytes; B1 without PCR control using whole testis total RNA; B2 - PCR control using no biological sample. M - 100 Base Pair Ladder (New England Biolabs). (b) Using forward *PDS1* primer and reverse *PDHA1* specific primer. PL - patient lymphocytes; PF - patient fibroblasts; C - control lymphocytes; B2 - PCR control using no biological sample. M - 100 Base Pair Ladder (New England Biolabs).

**Fig. 2:**
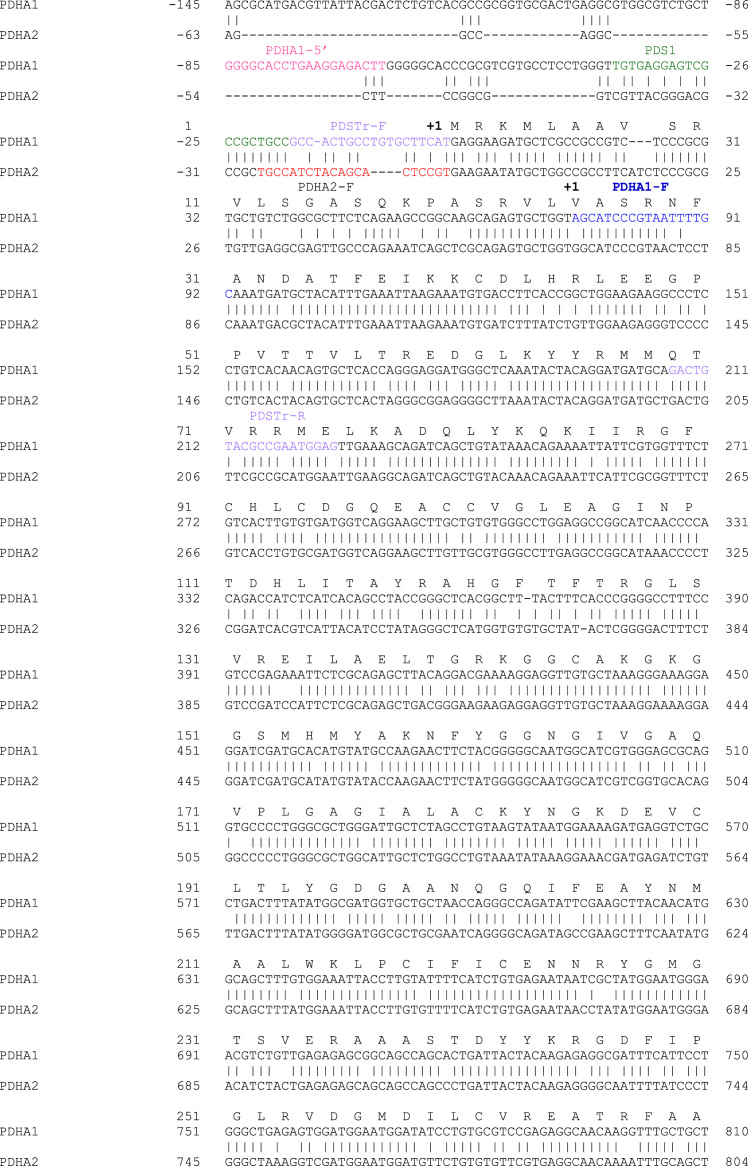

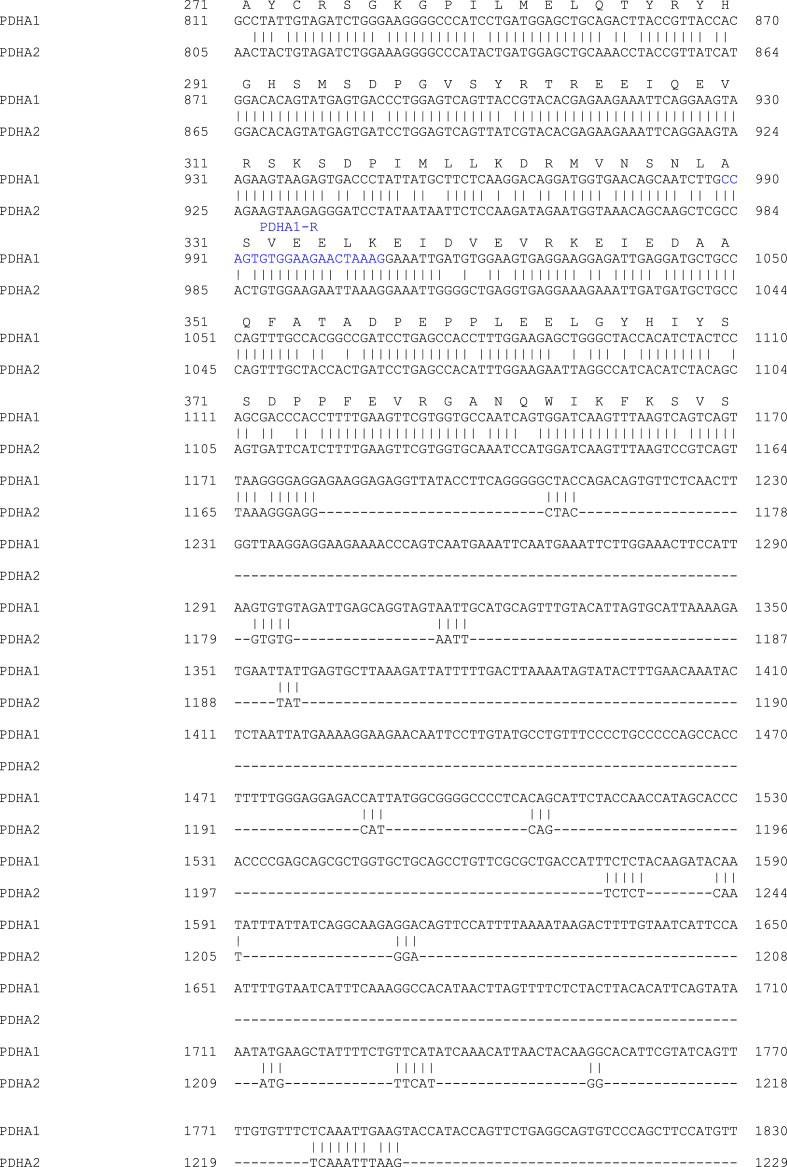

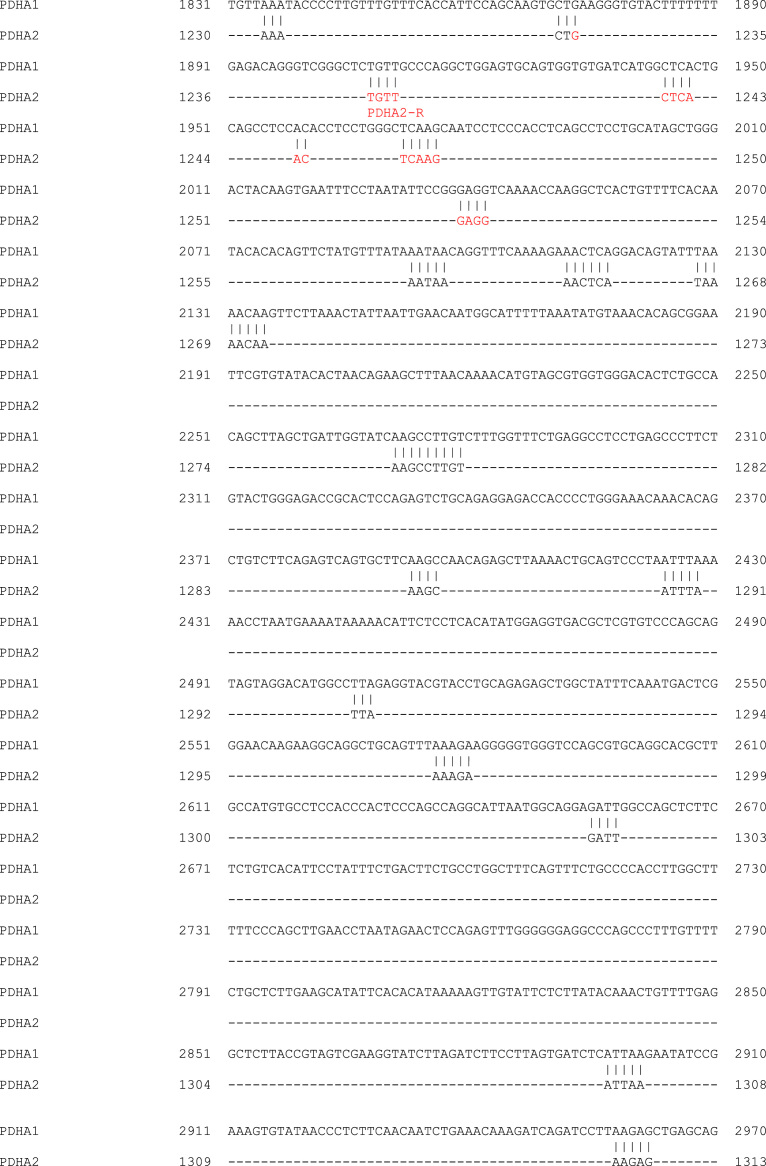

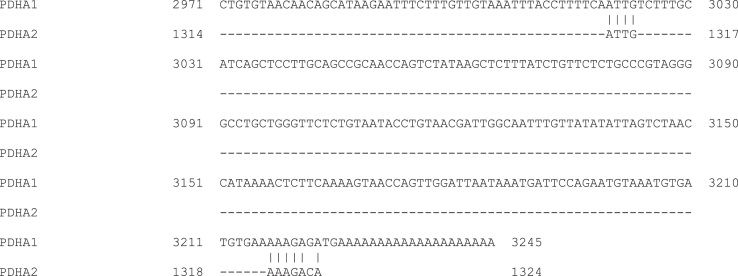
Alignment of *PDHA1* and *PDHA2* cDNA sequences and primers’ localization.

**Fig. 3:**
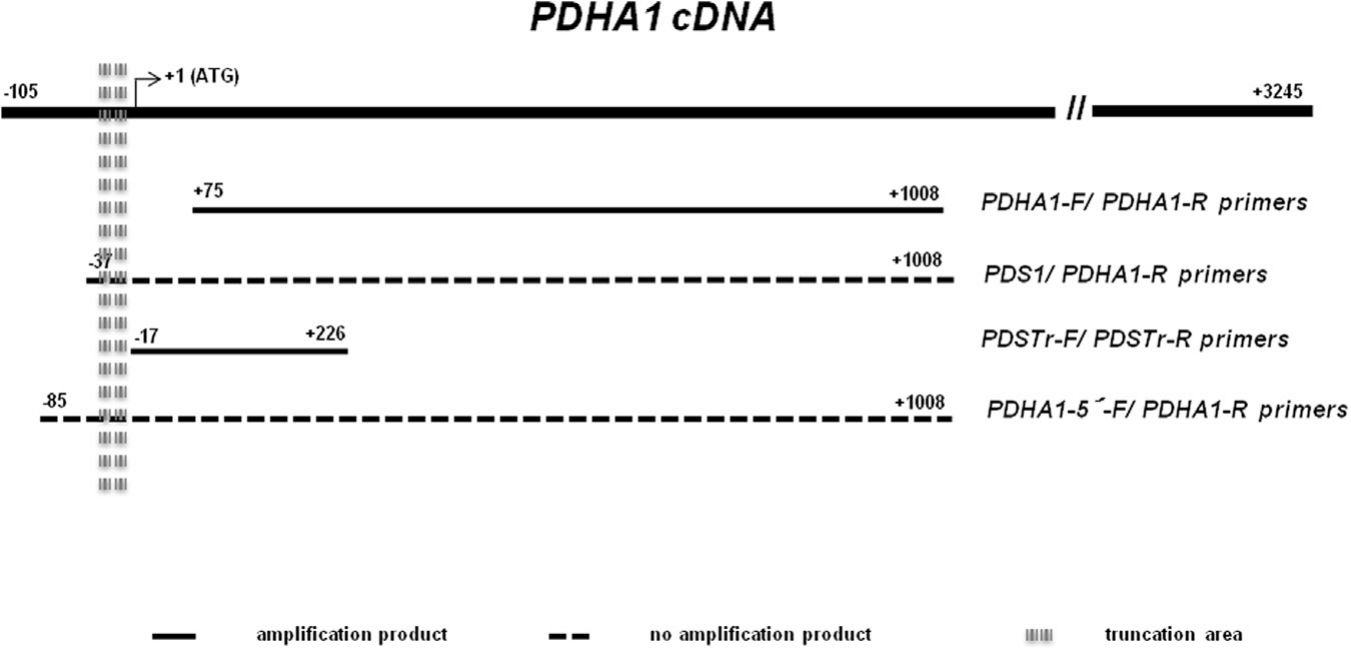
Schematic representation of the *PDHA1* mRNA sequence showing the amplified *versus* non-amplified products in the RT-PCR analysis with the corresponding localization of the forward primers (PDHA1-5′, PDS1, PDSTrF, PDHA1F) and reverse primers (PDHA1R and PDSTrR), as well as the identification of the predicted truncation point.

**Fig. 4:**
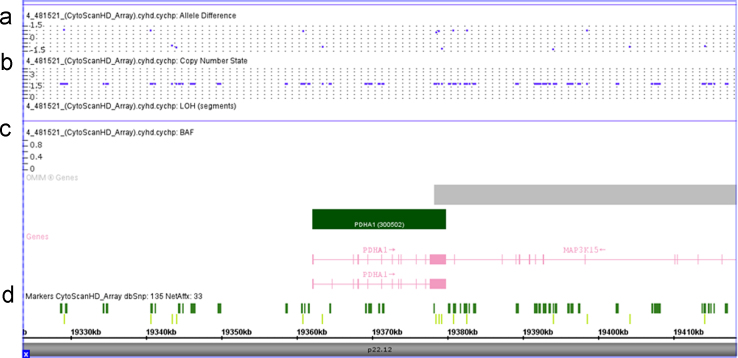
Detailed view of the *PDHA1* region on chromosome X. (a) Allele difference and (b) copy number state showing absence of big deletions involving the gene. (c) OMIM genes: *PDHA1* (dark green horizontal bar) and *MAP3K15* (gray horizontal bar). Intron - horizontal pink lines; Exon - vertical pink bars. (d) Markers present in *PDHA1* region. Dark green - non-polymorphic probes; Light green - SNP, single nucleotide polymorphism. (For interpretation of the references to color in this figure legend, the reader is referred to the web version of this article).

Table 1 shows the primers used for the amplification of the analyzed genes, according to the used methodology. Fig. 1 presents the results of *PDHA1* and *PDHA2* gene expression in somatic cells of the individuals under study and in controls. Fig. 2 displays the alignment of *PDHA1* and *PDHA2* mRNAs showing that the specific primers were designed to anneal to regions with null or very low homology between the two genes, thus proving the simultaneous presence of both transcripts. Fig. 3 depicts the scheme of *PDHA1* mRNA with the localization of all the primers used to prove the presence of the 5′UTR truncated *PDHA1* mRNA detected in the family samples, and to localize the truncation point. Table 2 and Fig. 4 show the results of the two different methodologies used to evaluate *PDHA1* gene copy number: quantitative real time PCR (Table 2) and microarray analyses (Fig. 4).

## 2 Experimental design, materials and methods

### 2.1 Sample preparation

Lymphocytes were isolated from three independent peripheral blood samples obtained from the index case and her parents and brother, as well as from control individuals.

Patient׳s fibroblast cultures were established from a diagnostic skin biopsy and grown under standard conditions.

Positive controls for *PDHA2* gene expression were obtained from two different sources; a commercially available human testis total RNA sample (Clontech Laboratories Inc., Mountain View, CA, USA) and human testis specimens from eight cases requiring open testicular biopsy for the retrieval of testicular sperm for intracytoplasmic sperm injection [3].

### 2.2 Nucleic acids preparation

Genomic DNA, total RNA and cDNA were prepared according to standard methods and described in [1].

### 2.3 PCR of genomic DNA and cDNA

Amplification of the 11 individual exons of the *PDHA1* gene and related intron–exon boundaries were amplified using primers already published [4]. *PDHA1* and *PDHA2* cDNAs were amplified under conditions previously described [5] and using primers listed in Table 1, which were designed to annealing to regions displaying no homology between transcripts [6].

### 2.4 Evaluation of PDHA1 and PDHA2 expression and PDHA1 gene dosage

*PDHA1* and *PDHA2* transcriptional levels were evaluated by quantitative real time RT-PCR under conditions previously described [1].

The copy number of *PDHA1* gene was evaluated by two methods, quantitative real time PCR and microarray analysis, as previously described [1].

## Figures and Tables

**Table 1:** List of primers used in this study.

**Primer**	**Sequence**		**Position**
**cDNA amplification**			

***PDHA1*****messenger**			
PDHA1-F	5′–AGCATCCCGTAATTTTGC–3′		+75 to +92
PDHA1-R	5′–CTTTAGTTCTTCCACACTGG–3′		+989 to +1008
PDHA1-5’-F	5′–GGGCACCTGAAGGAGACTT–3′		−85 to −66
PDS1	5′–TGTGAGGAGTCGCCGCTGCC–3′		−37 to −18
PDSTr-F	5′–GCCACTGCCTGTGCTTCAT–3′		−17 to +2
PDSTr-R	5′–ACTCCATTCGGCGTACAGTCT–3′		+207 to +226

***PDHA2*****messenger**			
PDHA2-F	5′–TGCCATCTACAGCACTCCGT–3′	−27 to −8	
PDHA2-R	5′–CCTCCTTGAGTTGAGAACAC–3′	+1235 to +1254	

***PDHX*****messenger**			
PXF2	5′–CTGCTGCGTTATCTTGTGGGCT–3′	+37 to +58	
PXW2	5′–TGAGTGAATGTGCCCACTGCATTG–3′	+812 to +835	
PXP2	5′–CAATGCAGTGGGCACATTCACTGA–3′	+812 to +835	
PXR2	5′–TAACAACTACTGAATCAACTAAGC–3′	+2060 to +2083	

**Genomic DNA amplification**			

***PDHA1*****gene**			
PDHA1-P1-F	5′–CCCTTGTTGCTTTGGTGTTT–3′	4383 to 4403	
PDHA1-P1-R	5′–AGATTGCTCTGCTGACTACCG–3′	4762 to 4784	
PDHA1-P2-F	5′–TGAGCATGCTGCTAATCTTCA–3′	4642 to 4682	
PDHA1-P2-R	5′–CGGCGTGACAGAGTCGTAAT–3′	5114 to 5133	
PDHA1-P3-F	5′–CTGGACGCCGTTCTGGTT–3′	4966 to 2983	
PDHA1-P3-R	5′–GCGGAGGCGAAGTAAAGG–3′	4323 to 4340	
PDHA1-P4-F	5′–TGCTTCATGAGGAAGATGCT–3′	5140 to 5159	
PDHA1-P4-R	5′–AGGGTGCTGTTTGAACGAAG–3′	5526 to 5645	

***PDHA2*****gene**			
PDHA2-A-F	5′–GAGTAAGGAAAAGTGGAATGTCA–3′	−841 to −819	
PDHA2-A-R	5′–ATCCTGCTCCATAATGTGCC–3′	−200 to −181	
PDHA2-B-F	5′–GCCATCAGGATAAATGTGGC–3′	−657 to −638	
PDHA2-B-R	5′–CCCTTTTCCCTGTTAAACCC–3′	−322 to −303	
PDHA2-C-F	5′–AACTCTCAGAACTCTCATGTGCC–3′	−415 to −393	
PDHA2-C-R	5′–ACGGAGTGCTGTAGATGGCA–3′	−27 to −8	
PDHA2-D-F	5′–CAGGACCTGCCTCTATCACC–3′	−142 to +123	
PDHA2-D-R	5′–AAACCGCGAATGAATTTCTG–3′	+244 to +263	
PDHA2-F-F	5′–GCATGGAATTGAAGGCAGAT–3′	+212 to +231	
PDHA2-F-R	5′–CCTCCTTGAGTTGAGAACAC–3′	+1298 to+1317	

***PDHX*****gene**			
PX1F	5′–AGAGACCTAAAGGCACCGCT–3′	+5414 to +5433	
PX1R	5′–AAGCAGGCCCTCAATCATAA–3′	+5751 to +5770	
PX2F	5′–TGGGAATCTTTTAGACTTTGGA–3′	+20,144 to + 20,165	
PX2R	5′–TGCTGAACCCAGAAAACCTT–3′	+20,531 to + 20,550	
PX3F	5′–CAACCCAGAAATAGCTACGGA–3′	+36,259 to + 36,279	
PX3R	5′–CACATTAAAAATAAGGAGGCAAAA–3′	+36,557 to + 36,581	
PX4F	5′–TGCAGTCATGGGGTTTTACTT–3′	+46,205 to + 46,225	
PX4R	5′–ACAGCAACTTCCTACGTGATG–3′	+46,549 to +46,570	
PX5F	5′–GTGACCATCTGTGGGAGTCA–3′	+49,159 to +49,173	
PX5R	5′–TTATTCAGAAAACAACTCTTGCAT–3′	+49,549 to +49,573	
PX6F	5′–TCACCTGCGTTTTCTGAAAGT–3′	+55,435 to + 55,456	
PX6R	5′–GTGAGCCAAGATTGTGCCAT–3′	+55,779 to +55,798	
PX7F	5′–TTCCACTTGTGGTTTAACGGA–3′	+58,968 to +58,988	
PX7R	5′–TTTCCTCTAGCACAAATATACCCA–3′	+59,294 to +59,318	
PX8F	5′–ACAAGTTTGAAGTTGTAATGGTCA–3′	+66,918 to +66,941	
PX8R	5′–GAGGGAGATCAAACGATAGGA–3′	+67,178 to +67,198	
PX9F	5′–TTTTTCTGTAACCGCCTTGG–3′	+73,376 to +73,395	
PX9R	5′–TCTCCCCTTCACACACACAA–3′	+73,700 to +73,719	
PX10F	5′–GGTAACAAAATCAAATCAAGGCA–3′	+81,064 to +81,085	
PX10R	5′–TTCAGATAAATGAAAGGCTGACA–3′	+81,315 to +81,337	
PX11F	5′–ACGGAAAGGGGACTTTGATT–3′	+83,725 to +83,744	
PX11R	5′–TTGAGGACTAGGCAAGTCGG–3′	+84,031 to +84,050	

***PDHA2*****gene methylation analysis**			
CpGI-M-F	5′–ATAAATTAGTTAGTTTAGGTTGCGT–3′	−188 to −164	
CpGI-M-R	5′–ATAACGTCATTTAAAAAATTACGAA–3′	+74 to +98	
CpGI-U-F	5′–ATAAATTAGTTAGTTTAGGTTGTGT–3′	−188 to −64	
CpGI-U-R	5′–ATAACATCATTTAAAAAATTACAAA–3′	+74 to +98	
CpGII-F	5′–TGGAATTGAAGGTAGATTAGTTGTATAAAT–3′	+205 to+234	
CpGII-R	5′–ATACCATTACCCCCATAAAAATTCT–3′	+406 to +431	

**Gene dosage analysis**			

***PDHA1*****gene**			
PDHA1-exon7F	5′–AGGAGGCCTTTCTGTGCTTT–3′	11,341 to 11,359	
PDHA1-exon7R	5′–CGGCCCCACCACAGGGTTCCT–3′	11,616 to 11,636	

***PAH*****gene**			
PAH-exon1F	5′–GCTTTACTGTGCGGAGATCACCAC–3′	5315 to 5339	
PAH-exon1R	5′–CTTATGAAACCAGGAAGCAC–3′	5606 to 5625	

**Table 2:** Calculations for determining by qPCR the copy number of *PDHA1* gene using as reference the autosomal *PAH* gene.

***PDHA1*****gene**				
Sample	Ave ΔCt	ΔΔCt	RQ (2^-ΔΔCt^)	Copy # (2×RQ)
Patient	0.26	0.91	0.5	1
Control Female 1	−0.65	0	1	2
Control Female 2	−0.33	0.32	0.8	2
Control Female 3	−0.59	0.06	0.9	2
Control Male 1	0.93	1.58	0.3	1
Control Male 2	−0.23	0.42	0.7	1
Control Male 3	−0.01	0.64	0.6	1
