# Supplementary material for: Data supporting the co-expression of PDHA1 gene and of its paralogue PDHA2 in somatic cells of a family
Source: Data Brief. 2016 Aug 20;9:68–77. doi: 10.1016/j.dib.2016.08.029 (PMC5021711; doi:10.1016/j.dib.2016.08.029)
Supplement: Supplementary file 1 — Supplementary material [file mmc1.doc]

The authors do declare they have no Conflict of Interest
